# A systematic review and meta-analysis of incidence and spatiotemporal trends of snakebites in Iran

**DOI:** 10.1371/journal.pntd.0013603

**Published:** 2025-10-14

**Authors:** Hossein Kargar Jahromi, Mohebat Vali, Nazanin Shafiei Jahromi, Mohammad Sadegh Moradi Sarcheshmeh, Amir Hossein Pourdavood, Zahra Moradi Kouchi, Zahra Yazdansetad, Ahmadreza Eidi, Hamed Delam

**Affiliations:** 1 Research Center for Non-Communicable Disease, Jahrom University of Medical Sciences, Jahrom, Iran; 2 Department of Epidemiology, School of Health, Shiraz University of Medical Sciences, Shiraz, Iran; 3 Department of Microbiology, Ja.C, Islamic Azad University, Jahrom, Iran; 4 Nurse and Midwifery Faculty, Shahed University, Tehran, Iran; 5 Kerman University of Medical Sciences, Kerman, Iran; 6 Gerash University of Medical Sciences, Gerash, Iran; 7 Shiraz University of Medical Sciences, Shiraz, Iran; 8 Larestan University of Medical Sciences, Larestan, Iran; 9 Student Research Committee, Shiraz University of Medical Sciences, Shiraz, Iran; University of Liverpool, UNITED KINGDOM OF GREAT BRITAIN AND NORTHERN IRELAND

## Abstract

**Background and purpose:**

Snakebites are a neglected public health concern, particularly in tropical regions, causing significant morbidity and mortality. Despite Iran’s high snakebite burden, epidemiological data remain inconsistent. This systematic review and meta-analysis aim to provide estimates of snakebite incidence and geographical distribution across Iranian provinces.

**Methods:**

A comprehensive search was conducted in PubMed/MEDLINE, Scopus, Web of Science, Embase, Google Scholar, and Persian databases (Magiran, SID) up to February 2025. Observational studies reporting snakebite incidence in Iran were included. Two independent reviewers screened studies, extracted data, and assessed bias using the Newcastle-Ottawa Scale (NOS). A random-effects meta-analysis was performed, with heterogeneity evaluated via I². Meta-regression analyzed temporal trends.

**Results:**

Of 618 initially identified studies, 8 met the inclusion criteria. This meta-analysis found Iran’s overall snakebite incidence to be 31.89 cases per 100,000 population (95% CI: 16.58-47.20), with extreme regional variation (0.14-295.45). Males showed a significantly higher incidence (108.34) than females (66.79). Geographic analysis revealed the highest rates in southeastern (109.68) and southwestern (116.04) regions, and the lowest in northwestern (4.30) and northern (4.05) areas. Meta-regression indicated a significant temporal increase in incidence (β = 0.035, p < 0.001). High heterogeneity (I² ≥ 99.8%) suggests additional underlying factors influence snakebite distribution.

**Conclusion:**

Snakebite incidence in Iran exhibits marked geographical and gender disparities, with an upward temporal trend. These findings highlight the need for targeted prevention strategies, improved antivenom access, and enhanced surveillance in high-risk provinces.

## Author summary

**Why was this study done?:**

Snakebites are a serious but often overlooked health problem, especially in countries like Iran. However, getting a clear picture of how many people are bitten and where it happens most has been difficult due to inconsistent data. This study aimed to provide the first reliable nationwide estimates of snakebite incidence across Iran to help guide public health efforts.

**What did the researchers do and find?:**

We combined and analyzed the results of all relevant published studies on snakebites in Iran. Our key findings were:

- The national snakebite incidence is approximately 32 cases per 100,000 people each year.
- The risk is not evenly distributed. The southeastern and southwestern provinces (like Sistan-Baluchestan and Khuzestan) have alarmingly high rates, over 100 times greater than in northern provinces.
- Men are bitten at a significantly higher rate than women.
- The data shows that snakebites have been increasing over time across the country.

**What do these findings mean?:**

These results reveal a critical and growing health disparity within Iran. The high incidence in specific regions calls for urgent, targeted action. Public health officials should focus prevention programs, ensure adequate antivenom supplies, and strengthen medical training in the high-risk provinces we identified.

## Introduction

Snakebites represent a significant yet often overlooked public health issue, particularly in tropical and subtropical regions where frequent human-snake interactions occur. According to the World Health Organization (WHO), an estimated 5.4 million snakebites occur globally each year, resulting in 1.8 to 2.7 million cases of envenoming and 81,000–138,000 deaths annually [1]. These figures underscore the urgent need for increased awareness, improved medical interventions, and better data collection to address this neglected tropical disease (NTD). Snakebites disproportionately affect rural populations in low- and middle-income countries, where access to healthcare and antivenom is often limited [2].

The incidence of snakebites varies significantly across regions, with the highest rates reported in South Asia, Sub-Saharan Africa, and Southeast Asia [3]. The global incidence of snakebites is estimated at 69.4 cases per 100,000 population annually [4]. In the Americas, the average annual incidence of snakebites is approximately 57,500 cases, equivalent to 6.2 cases per 100,000 population. The mortality rate is around 370 yearly deaths, corresponding to 0.04 per 100,000 population [5]. In India alone, snakebites account for approximately 45,900 deaths annually, making it one of the most affected countries [6]. Similarly, in Sub-Saharan Africa, snakebites are a leading cause of morbidity and mortality, particularly among agricultural workers and children [3,7]. According to a report by the Iranian Ministry of Health and Medical Education, between 2001 and 2010, approximately 4,500–7,000 people were affected by snakebites annually. The mortality rate ranged from 3 to 9 yearly deaths, with an average annual incidence of 6.9 cases per 100,000 population during this period [8,9]. The geographical distribution of venomous snakes, coupled with socioeconomic factors such as poverty and inadequate healthcare infrastructure, exacerbates the impact of snakebites in these regions [10].

The clinical consequences of snakebites depend on the snake species, the amount of injected venom, and the medical intervention’s timeliness. Venomous snakes can be broadly categorized into two families: Viperidae (vipers) and Elapidae (cobras, kraits, and mambas), each producing distinct types of venom that cause varying symptoms [11]. Viper venoms often cause local tissue damage, coagulopathy, and hemorrhage, whereas elapid venoms are more likely to cause neurotoxicity and respiratory failure [12].One of the most significant challenges in managing snakebites is the scarcity of reliable epidemiological data. Many snakebite incidents go unreported, particularly in rural areas, where victims may rely on traditional medicine rather than seeking formal medical care [13].

Recently, there has been growing recognition of snakebites as an NTD, prompting calls for increased funding and research. In 2017, the WHO included snakebite envenoming on its list of NTD, marking a significant step toward global action [14]. However, much work remains to be done to reduce the incidence of snakebites and mitigate their impact on vulnerable populations. Various studies conducted in Iran reported inconsistent findings regarding the incidence of snakebites. To manage these discrepancies, this systematic review and meta-analysis aims to estimate the incidence and geographical distribution of snakebites across different provinces in Iran.

## Methods

### Study design

This study is a systematic review and meta-analysis of the incidence of snakebites in Iran. The review aims to synthesize available evidence on the epidemiology of snakebites, including incidence rates, geographical distribution, and temporal trends.

### Eligibility criteria

Studies were included based on the following criteria: Population: Studies reporting on human populations in Iran. Exposure: Snakebites, confirmed either clinically or through laboratory testing. Outcome: Incidence rates of snakebites (cases per 100,000 population) and geographical distribution. Study Design: Observational studies, including cross-sectional studies, cohort studies, and hospital-based studies. Case reports and reviews were excluded. Language: Studies published in English or Persian. Timeframe: No time limit was considered, and all articles published up to February 2025 were included. Gray literature, including conference and congress abstracts, was not reviewed. Articles that did not include a population defined for disease incidence were also excluded.

### Information sources

A comprehensive search was conducted across the following electronic databases: PubMed/MEDLINE, Scopus, Web of Science, Embase, Magiran (for Persian-language studies), Scientific Information Database (SID), and Google Scholar. Furthermore, reference lists from the included studies and relevant reviews were manually checked to identify additional eligible studies.

### Search strategy

The search strategy was developed in consultation with a medical librarian and included a combination of Medical Subject Headings (MeSH) terms and free-text keywords. The following search terms were used: (snake OR snake-bite OR snake-bites OR snakebites OR snakebite OR snakebite-envenoming OR snake-envenoming OR snakebite-envenomation OR snake-envenomation) AND (“Iran”). The “Incidence” was not used in the search strategy to increase search sensitivity. So, more articles could be retrieved, and eligible articles could be selected from among them. The search strategy was adapted for each database to account for differences in indexing and syntax. No filters were applied for study design or language during the initial search. The findings of this systematic review and meta-analysis are reported following the PRISMA guidelines. A PRISMA flow diagram was used to document the study selection process, including the number of studies identified, screened, included, and excluded.

### Study selection

The retrieved articles were initially entered into EndNote version 8. Then, considering the software’s ability to find duplicate articles, these articles were identified and removed. Two researchers also reviewed all articles entered into the library for duplicates. Two researchers independently screened titles and abstracts, reviewed full texts for eligibility, and resolved any disagreements through discussion or by consulting a third reviewer.

### Data extraction

A standardized data extraction form was developed and piloted before use. The following data were extracted from the included studies: Study characteristics: author, publication year, study period, and geographical location. Population characteristics: sample size and gender. Snakebite data: total number of snakebites and incidence rate.

### Risk of bias assessment

The methodological quality of included studies was assessed using the NOS for observational studies. Studies were rated based on three areas: Selection of study groups, Comparison of groups, and Outcome assessment [15,16]. Studies with a score of ≥6 out of 9 were considered high quality.

### Statistical data analysis

The primary outcome was the incidence of snakebites, expressed as cases per 100,000 population per year. If incidence rates were not directly reported, they were calculated using population data from the Iranian Statistical Center. Meta-analysis was performed using a random-effects model to account for heterogeneity between studies. Pooled incidence rates were reported with 95% confidence intervals (CIs). Heterogeneity was measured using the I² statistic (25%, 50%, 75% for low, moderate, high). Sensitivity analyses excluded studies with high bias or small samples to test robustness. Publication bias was checked with funnel plots and Egger’s test if over 10 studies were included. Meta-regression analyzed pooled data to assess disease progression over time. All statistical analyses were conducted using CMA software (version 2) and R software version 4.4.2. Descriptive statistics were used to summarize study characteristics and demographic data. Incidence rates were pooled using the Restricted maximum likelihood (REML)method, and heterogeneity was assessed as described above. A p-value of <0.05 was considered statistically significant.

### Ethical considerations

This study utilized publicly available data and did not involve direct contact with human subjects. Ethical approval was not required.

## Results

### Characteristics of studies

Initially, 618 studies were retrieved, of which 227 were in Scopus, 177 in Web of Science, 21 in PubMed, 61 in Embase, 120 in Google Scholar, and 12 in Persian databases.

**Fig 1:**
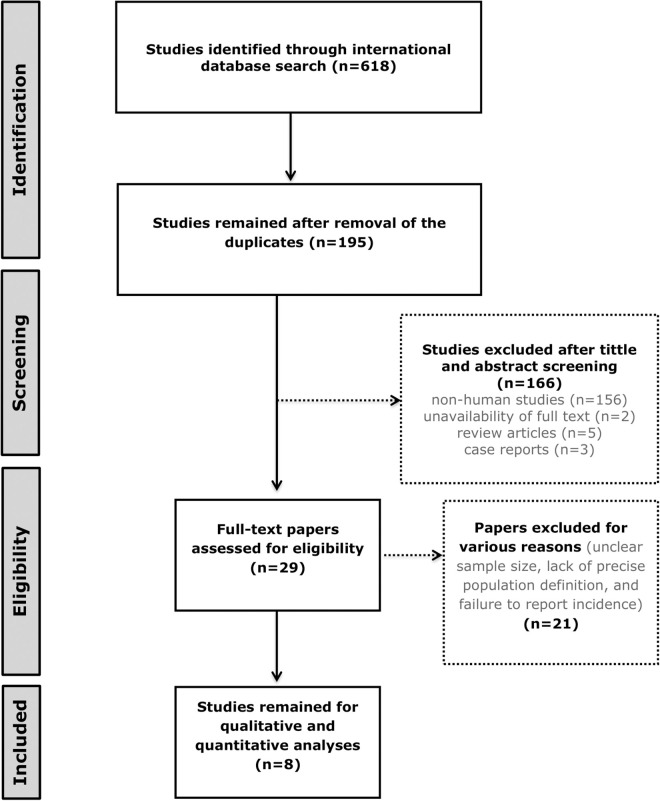
Study protocol.

After removing duplicate articles, 195 articles remained, of which 166 were excluded due to reasons such as non-human studies (n = 156), unavailability of full text (n = 2), review articles (n = 5), and case reports (n = 3). The remaining 29 articles were selected for full text review, of which 21 studies were excluded due to review articles, unclear sample size, lack of precise population definition, and failure to report incidence. Finally, 8 articles were selected for data extraction and statistical analysis [9,17–23] (Table 1 and Fig 1). In the supplementary section, the search strategy, the number of retrieved articles and reasons for exclusion of studies (S1 Table) are reported.

**Table 1:** Incidence rate per 100,000 population by region, gender, and time period.

Study name	Region	Time Period	Gender	Incidence per 100,000
Dehghani, 2014	East Azerbaijan	2009-2010	Both	1.30
Dehghani, 2014	West Azerbaijan	2009-2010	Both	3.10
Dehghani, 2014	Ardabil	2009-2010	Both	3.00
Dehghani, 2014	Isfahan	2009-2010	Both	3.10
Dehghani, 2014	Alborz	2009-2010	Both	0.24
Dehghani, 2014	Ilam	2009-2010	Both	4.00
Dehghani, 2014	Bushehr	2009-2010	Both	22.60
Dehghani, 2014	Tehran	2009-2010	Both	0.30
Dehghani, 2014	Chaharmahal and Bakhtiari	2009-2010	Both	6.50
Dehghani, 2014	South Khorasan	2009-2010	Both	32.50
Dehghani, 2014	Razavi Khorasan	2009-2010	Both	0.30
Dehghani, 2014	North Khorasan	2009-2010	Both	7.80
Dehghani, 2014	Khuzestan	2009-2010	Both	17.60
Dehghani, 2014	Zanjan	2009-2010	Both	8.20
Dehghani, 2014	Semnan	2009-2010	Both	114.60
Dehghani, 2014	Sistan - Baluchestan	2009-2010	Both	29.60
Dehghani, 2014	Fars	2009-2010	Both	2.60
Dehghani, 2014	Qazvin	2009-2010	Both	8.30
Dehghani, 2014	Qom	2009-2010	Both	1.80
Dehghani, 2014	Kurdistan	2009-2010	Both	4.60
Dehghani, 2014	Kerman	2009-2010	Both	21.40
Dehghani, 2014	Kermanshah	2009-2010	Both	37.20
Dehghani, 2014	Kohgiluyeh and Boyer Ahmad	2009-2010	Both	0.14
Dehghani, 2014	Golestan	2009-2010	Both	7.60
Dehghani, 2014	Gilan	2009-2010	Both	2.40
Dehghani, 2014	Lorestan	2009-2010	Both	10.80
Dehghani, 2014	Mazandaran	2009-2010	Both	2.90
Dehghani, 2014	Markazi	2009-2010	Both	1.80
Dehghani, 2014	Hormozgan	2009-2010	Both	31.00
Dehghani, 2014	Hamedan	2009-2010	Both	0.80
Dehghani, 2014	Yazd	2009-2010	Both	3.00
Dehghani, 2014	Iran	2009-2010	Both	6.90
Dehghani, 2014	Iran	2002	Both	8.40
Dehghani, 2014	Iran	2003	Both	9.10
Dehghani, 2014	Iran	2004	Both	8.60
Dehghani, 2014	Iran	2005	Both	8.30
Dehghani, 2014	Iran	2006	Both	9.00
Dehghani, 2014	Iran	2007	Both	6.90
Dehghani, 2014	Iran	2008	Both	6.60
Dehghani, 2014	Iran	2009	Both	6.60
Dehghani, 2014	Iran	2010	Both	6.20
Dehghani, 2014	Iran	2011	Both	4.50
Dehghani, 2022	Lordegan - Chaharmahal and Bakhtiari	2019-2020	Both	93.76
Ebrahimi, 2018	Haji-Abad city - Hormozgan	2012-2016	Both	295.45
Kassiri, 2022	North of Sistan - Baluchistan	2019-2022	Both	176.40
Kassiri, 2019	Khorram-shahr - Khuzestan	2013-2017	Both	76.63
Kassiri, 2018	Dezful - Khuzestan	2013	Both	16.53
Kassiri, 2018	Shush - Khuzestan	2013	Both	7.89
Kassiri, 2018	Gotvand - Khuzestan	2013	Both	4.62
Nejadrahim, 2019	Urmia - West Azerbaijan	2012-2014	Both	5.77
Dehghani, 2014	Iran	2009-2010	Male	4.56
Dehghani, 2014	Iran	2009-2010	Female	2.34
Dehghani, 2022	Lordegan - Chaharmahal and Bakhtiari	2019-2020	Male	64.15
Dehghani, 2022	Lordegan - Chaharmahal and Bakhtiari	2019-2020	Female	123.37
Kassiri, 2022	North of Sistan - Baluchistan	2019-2020	Male	251.93
Kassiri, 2022	North of Sistan - Baluchistan	2019-2020	Female	100.87
Kassiri, 2019	Khorram-shahr - Khuzestan	2013-2017	Male	112.70
Kassiri, 2019	Khorram-shahr - Khuzestan	2013-2017	Female	40.57

### Overall incidence rate

**Fig 2:**
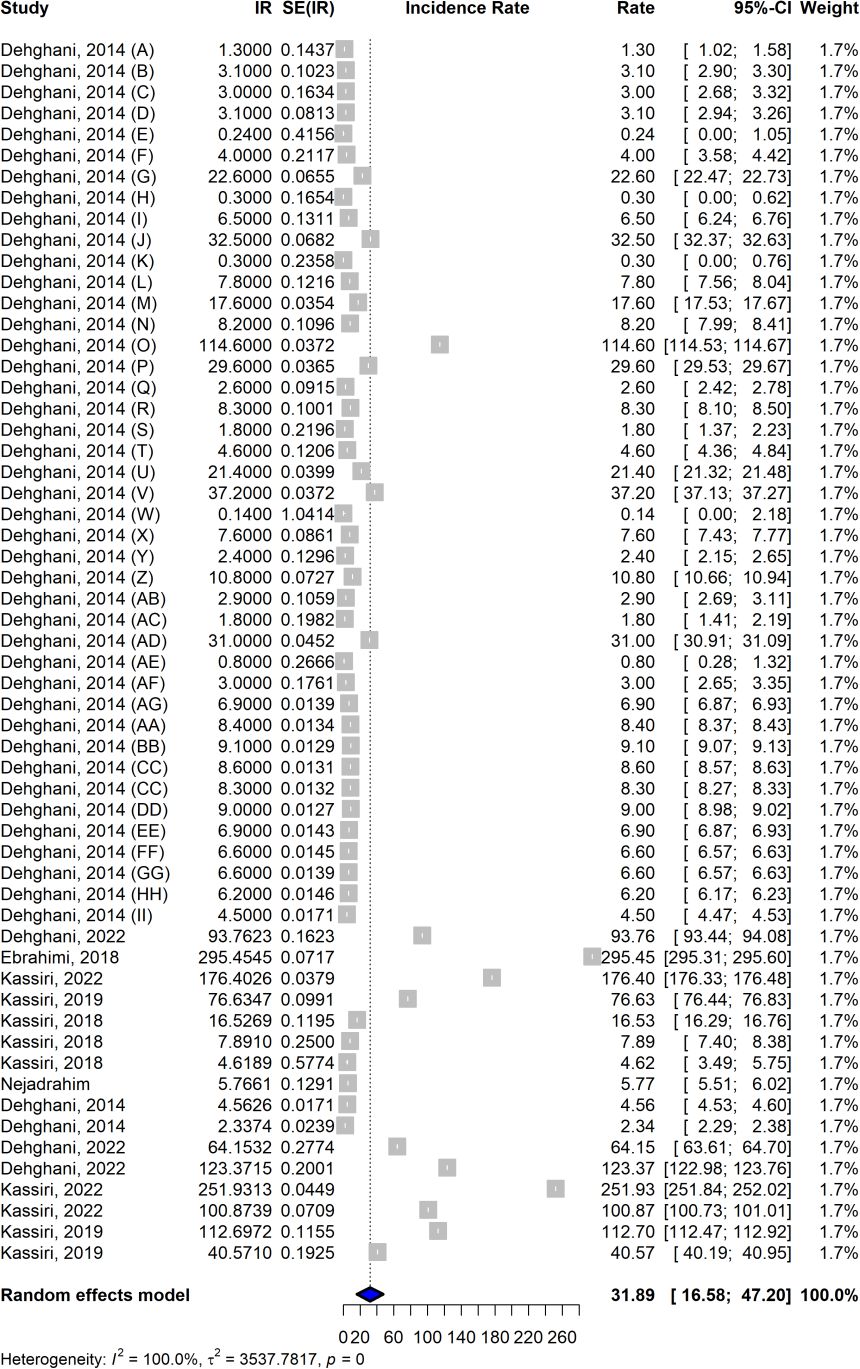
The overall incidence rate of snakebite in Iran.

The incidence of snakebites in Iran was estimated to be 31.89 (95% CI: 16.58,47.20) cases per 100,000 population. The random-effects model estimated a wide range of incidence rates, from 0.14 to 295.45 cases per 100,000 population, highlighting significant variability across regions and studies. The highest reported incidence was 295.45 per 100,000 in the Ebrahimi study, while the lowest was 0.14 per 100,000 in the Dehghani study (Fig 2).

### Subgroup analysis

**Fig 3:**
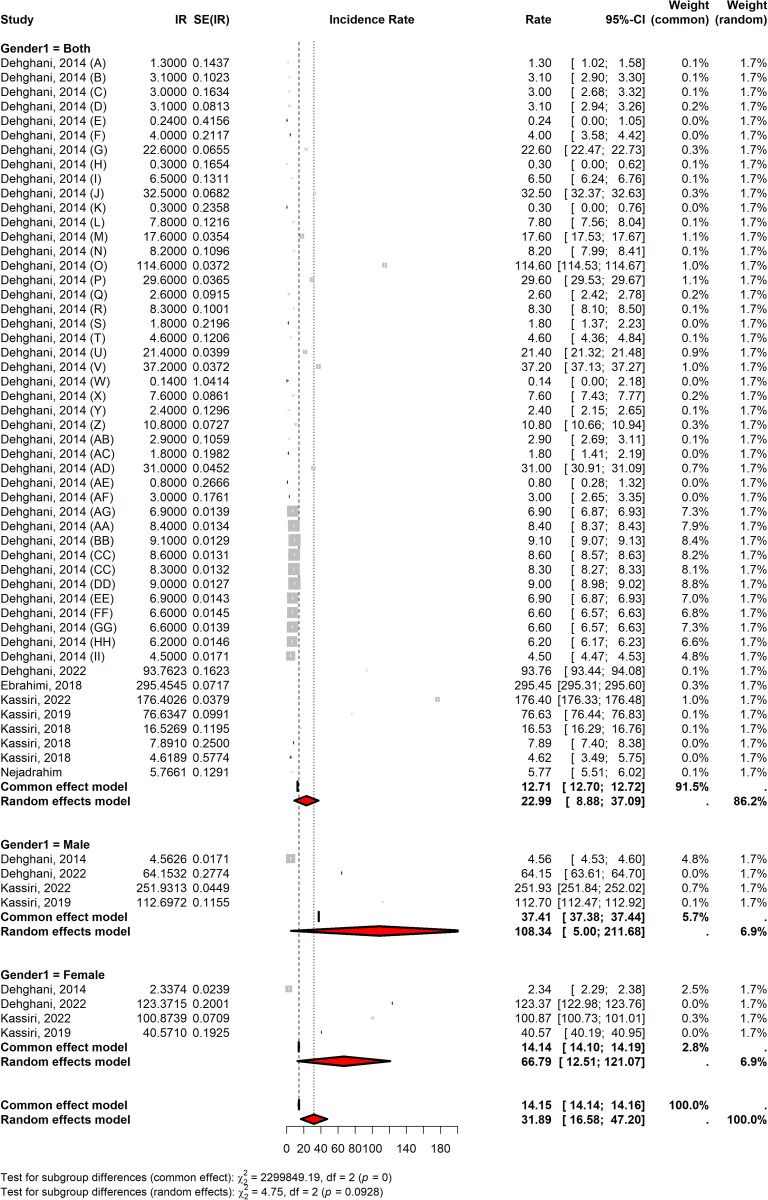
The overall incidence rate of snakebite in Iran by gender.

The subgroup analysis showed substantial variations in snakebite incidence rates across different demographic and geographic categories in Iran. When stratified by gender, males showed significantly higher incidence rates (108.33 per 100,000; 95% CI: 5.00–211.67) compared with females (66.78 per 100,000; 95% CI: 12.50–121.07). The overall incidence rate for both genders combined was 22.98 per 100,000 (95% CI: 8.88–37.08) (Fig 3). Extreme heterogeneity (I² = 100%) was observed across all gender subgroups, with statistically significant differences (P-value<0.001), suggesting pronounced variability in snakebite risk between males and females. Geographic analysis demonstrated marked regional disparities in snakebite incidence. The highest incidence rates were observed in the southeast (109.68 per 100,000; 95% CI: 0.00–292.44) and southwest (116.04 per 100,000; 95% CI: 29.74–202.33) regions, while the lowest rates were reported in the northwest (4.30 per 100,000; 95% CI: 1.05–7.54) and north (4.05 per 100,000; 95% CI: 0.00–11.40). The central, east, and west regions exhibited intermediate rates, ranging from 32.50 to 37.51 per 100,000. Heterogeneity remained consistently high across all geographic subgroups (I² ≥ 99.80%), with significant (P-value<0.001), indicating substantial variation in snakebite incidence between regions (Table 2). The subgroup analysis showed that the highest incidence was observed in Semnan (114.6 per 100,000), Sistan-Baluchestan (64.45 per 100,000), and Hormozgan (41.6 per 100,000) provinces, respectively; while the lowest incidence rate was observed in Alborz (0.2 per 100,000), Tehran (0.3 per 100,000), and Razavi Khorasan (0.3 per 100,000) provinces.

**Table 2:** Subgroup Analysis of Snakebite Incidence Rates in Iran by Gender and Geographic area.

Variables	Number of studies	Incidence rate per 100,000 (95% CI)	I^2^ (%)	P-value
**Gender**				<0.001
Both	50	22.98 (8.88, 37.08)	100	
Male	4	108.33 (5.00, 211.67)	100	
Female	4	66.78 (12.50,121.07)	100	
**Area**				<0.001
Iran	13	6.76 (5.67, 7.86)	100	
Northwest	6	4.94 (2.59, 7.29)	99.80	
North	3	4.30 (1.05, 7.54)	99.90	
Northeast	2	4.05 (0.00, 11.40)	99.90	
West	5	11.48 (0.00, 24.47)	100	
Central	11	37.51 (7.46, 67.55)	100	
East	1	32.50 (32.36, 32.63)	–	
Southwest	9	33.25 (8.54, 57.96)	100	
South	3	109.68 (0.00, 292.44)	100	
Southeast	5	116.04 (29.74, 202.33)	100	

I^2^: heterogeneity measure.

### The meta-regression results

**Fig 4:**
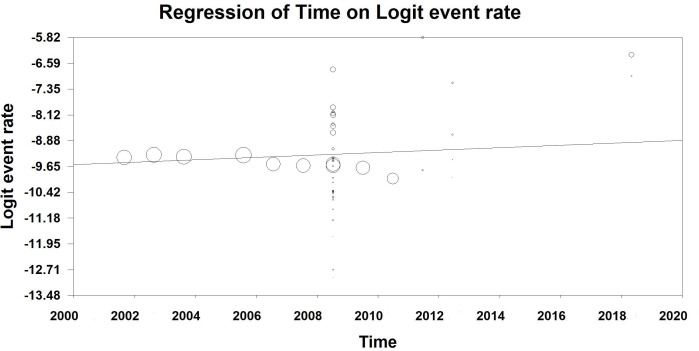
Results of meta-regression of the relationship between the incidence rate of snakebites in Iran and time.

The meta-regression results show that there is a significant positive relationship (y = -80.57 + 0.035*time) between the incidence of snakebites and time, such that the incidence of snakebites has increased over time (P-value<0.001) (Fig 4).

### Sensitivity analysis

**Fig 5:**
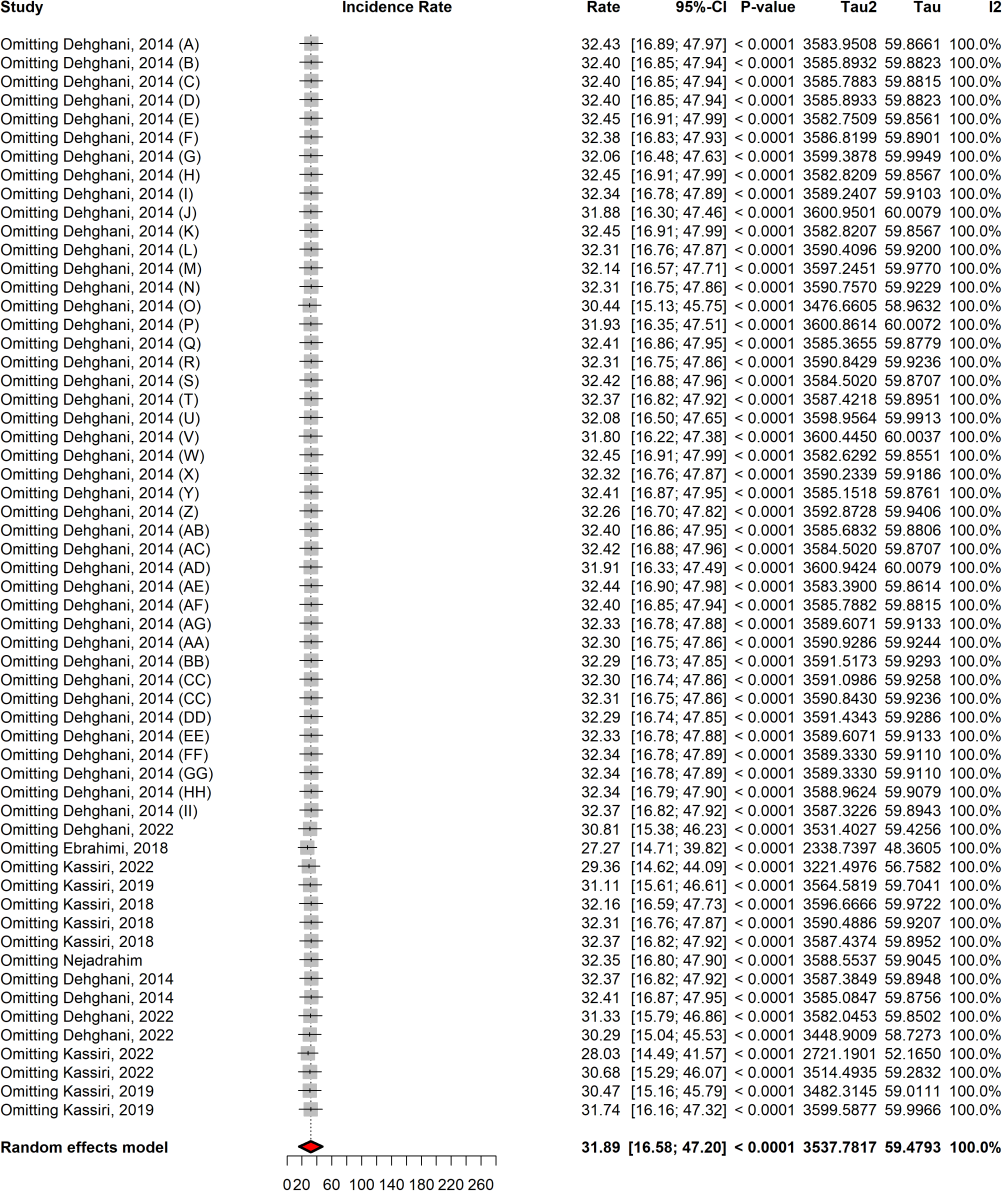
Results of sensitivity analysis of snakebite incidence rate in Iran.

The sensitivity analysis evaluated the stability of the meta-analysis by sequentially removing individual studies to determine their effect on the overall incidence rate. The pooled incidence rate remained relatively stable across all sensitivity tests, with minor fluctuations around ~31.89 cases per 100,000 population (95% CI: 16.58–47.20). No single study significantly altered the pooled estimate, confirming the robustness of the meta-analysis (Fig 5).

### Publication bias

**Fig 6:**
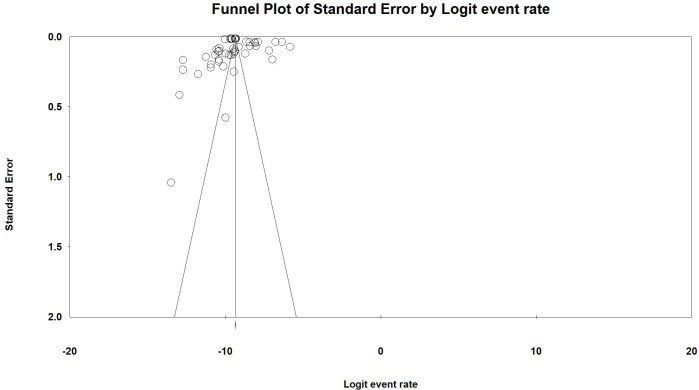
Funnel plot.

The funnel plot was visually inspected for potential publication bias by plotting the standard error against the logit-transformed event rates. While some asymmetry was observed in the distribution of studies, Egger’s regression test did not reveal statistically significant evidence of publication bias (Egger’s test: P-value = 0.271). This suggests that the observed asymmetry may be attributable to factors other than publication bias, such as substantial heterogeneity across studies or true variations in snakebite incidence across different regions in Iran (Fig 6).

## Discussion

The findings of this meta-analysis reveal important patterns in snakebite epidemiology in Iran that warrant comparison with global data. When examining the overall incidence rate of 31.89 cases per 100,000 population, we observe that Iran occupies an intermediate position between high-incidence tropical countries and low-incidence temperate regions. This becomes particularly evident when compared with data from South Asia, where Sri Lanka shows exceptionally high rates of 398 per 100,000 [24]. A study in Bangladesh reported a snakebite incidence rate of approximately 623 per 100,000 person-years, attributing this high rate to the geographical location and natural environmental conditions suitable for snakes [25]. The lower incidence in Iran compared to these regions may be attributed to several factors, including differences in snake species distribution, population density, and agricultural practices. However, Iran’s incidence remains substantially higher than European countries (1.06 per 100,000) as reported by Chippaux [26]. The geographical distribution of cases within Iran shows striking parallels with global patterns of snakebite epidemiology. The high incidence provinces of Semnan (114.6 per 100,000), Sistan-Baluchestan (64.45), and Hormozgan (41.6) share ecological characteristics with other high-risk regions worldwide. These areas are characterized by arid and semi-arid climates that favor the proliferation of medically important viper species, particularly *Echis carinatus* (saw-scaled viper) and *Macrovipera lebetina* (Levantine viper), similar to the epidemiological patterns observed in neighboring Pakistan [27]. The ecological factors contributing to this pattern include the availability of suitable habitats for these snake species in rocky foothills and agricultural areas, combined with human activities that increase exposure risk. Conversely, the low incidence observed in Tehran (0.3 per 100,000) and Alborz (0.2) provinces mirrors the urban protection effect seen in other parts of the world, where urbanization reduces both snake habitats and human-snake encounters [28]. The significant gender disparity in snakebite incidence, with males showing 1.6 times higher rates, represents a consistent finding across snakebite epidemiology literature worldwide. The results of a study in El Salvador showed that the ratio of snakebite cases in men to women was 1.59 [29]. In most countries, agriculture is done by men; many of these people tend to cultivate and work barefoot, especially in rice fields, a behavior that probably leads to a higher incidence of snakebites in men [30]. Men also tend to work outdoors, contributing to their susceptibility to snakebites. Bites occurring on the upper limbs are usually intentional, and men seem to be more likely to provoke snakes than women [31].

This study showed that the incidence of snakebites has been increasing at a gentle rate over time. While another study showed that in some areas of Ghana, the incidence of the disease has been decreasing, in some areas of this country, high rates of snakebites are still reported [32]. A study in California found that the incidence of snakebites over 20 years depended on rainfall and drought patterns. Snakebite rates decreased after drought and increased after rainfall [33]. There are various reasons to justify the increase in the incidence of snakebites in recent years; it seems that more people who have been bitten are seeking health care and accurate registration of cases. The development of agriculture and animal husbandry in the country, especially in rural areas, and the development of villages in terms of construction and entering the snake habitat have increased the exposure to bites in people.

### Strengths and limitations

The inclusion of studies without time restrictions enhances the temporal scope of the analysis, allowing for the examination of long-term trends. Additionally, using a random-effects model accounts for heterogeneity across studies, providing a more robust pooled estimate of snakebite incidence. The subgroup analyses by gender and geographic region offer important epidemiological distinctions, highlighting high-risk populations and areas that require targeted interventions. Furthermore, sensitivity analysis confirmed the stability of the results, and Egger’s test suggested no significant publication bias, reinforcing the reliability of the findings.

However, several limitations should be acknowledged. One of the study’s main limitations was the failure to calculate the incidence rate based on person-time, making the incidence rate calculations very complicated due to the lack of access to this number. Additionally, the gray literature exclusion and non-Persian/English studies might have omitted relevant data, particularly from local reports or unpublished research. Another limitation is the lack of detailed ecological and behavioral data, such as snake species distribution, seasonal variations, and occupational risk factors, which could further explain the observed disparities in incidence. The meta-regression, while indicating a temporal increase in snakebites, cannot establish causality, as factors like improved reporting, climate change, or land-use alterations may contribute to this trend. Finally, the small number of included studies (n = 8) may limit the generalizability of the findings, emphasizing the need for more high-quality, population-based studies in underrepresented regions in Iran. A significant limitation of this study is the lack of data on mortality, long-term disability, and ecological factors. This lack limits the interpretation of our findings in several key ways. While we can describe the incidence and geographic distribution of reported snakebites, we cannot assess their ultimate public health severity. The inability to determine mortality rates means that the true rate of snakebite-related deaths in the study population remains unknown. Furthermore, without follow-up data on disability, our study underestimates the total burden of disease because it misses chronic consequences such as amputations, musculoskeletal complications, and psychological trauma, which have profound long-term impacts on victims’ lives and livelihoods. Finally, the exclusion of ecological and seasonal variables prevents our model from identifying high-risk temporal patterns or environmental conditions, thereby limiting the practical application of our findings for designing targeted prevention campaigns. Consequently, our results should be considered as a basic description of the occurrence of snakebites, not a comprehensive assessment of its full burden or the environmental drivers behind it.

## Conclusion

This meta-analysis reveals crucial insights into Iran’s snakebite epidemiology, demonstrating a national incidence rate of 31.89 cases per 100,000 population (95% CI: 16.58-47.20) with extreme geographical variation ranging from 0.14 to 295.45 cases across different regions. The analysis identified significantly higher incidence among males (108.34/100,000) compared to females (66.79/100,000), reflecting gender-based differences in exposure risks, while temporal trends showed a concerning annual increase in incidence (β = 0.035, p < 0.001). Geographically, Semnan (114.6/100,000), Sistan-Baluchestan (64.45/100,000), and Hormozgan (41.6/100,000) emerged as high-risk provinces, contrasting sharply with northern regions showing minimal cases. The extreme heterogeneity across studies suggests additional underlying factors, including ecological conditions, occupational hazards, and potentially climate-related influences, contribute to this complex epidemiological pattern. These findings underscore snakebite as a growing public health challenge in Iran that demands immediate, evidence-based interventions. Strategic responses should prioritize high-risk regions with targeted antivenom distribution and healthcare worker training, while gender-specific prevention programs should address occupational risks for male workers. The establishment of a national surveillance system could effectively monitor temporal trends and identify emerging hotspots, complemented by research investigating climate change impacts and human-snake conflict patterns. The demonstrated increasing incidence trend highlights the urgency of implementing comprehensive prevention and treatment strategies. This include integrating snakebite management into Iran’s primary healthcare system to mitigate future disease burden and protect vulnerable populations in high-risk regions and occupations. These measures would not only address current challenges but also establish a framework for ongoing monitoring and adaptive management of this significant public health issue.

### Recommendations

Based on the findings, several recommendations can be made to improve snakebite prevention and management in Iran. First, enhanced surveillance systems should be established to ensure accurate and standardized reporting of snakebite cases across all provinces, particularly in high-incidence areas such as Semnan, Sistan-Baluchestan, and Hormozgan. Integrating snakebite data into national health registries would facilitate real-time monitoring. Second, targeted public health interventions should be implemented, focusing on high-risk populations such as male agricultural workers, who exhibit significantly higher incidence rates. Community education programs on snakebite prevention, first aid, and the importance of seeking immediate medical care could reduce morbidity and mortality.

Furthermore, ecological and behavioral research should be prioritized to identify key risk factors, such as climate influences, land-use changes, and human-snake conflict patterns. Long-term studies assessing the impact of climate change on snakebite incidence could inform predictive models and adaptive public health strategies. Finally, policy advocacy is needed to recognize snakebite as an NTD in Iran, ensuring sustained funding and research attention. Multisectoral collaboration between the Ministry of Health, environmental agencies, and agricultural departments could address the root causes of human-snake encounters, such as habitat encroachment. By adopting these measures, Iran can mitigate the growing burden of snakebites and align with global efforts to reduce snakebite-related deaths and disabilities by 2030. The present study provides an important basis for understanding the epidemiology of snakebite in Iran, but its scope requires an acknowledgement of certain limitations to guide future research. The study analysis did not assess patient outcomes following snakebite, leaving a gap in knowledge about mortality rates and long-term disability burden, which are essential to quantify the true impact on health. Furthermore, the influence of ecological and seasonal factors remained unexplored, limiting the ability to predict high-risk periods or environments. To build a more comprehensive picture, future research should prioritize longitudinal studies that track clinical outcomes to determine mortality rates, prevalence of chronic complications, and subsequent socioeconomic consequences for affected individuals and families. At the same time, integrating climate data—including temperature, humidity, and rainfall patterns—with epidemiological records is critical to elucidating seasonal trends and ecological drivers of human-snake conflict.

## Supporting information

S1 Table List of screened and deleted articles. (DOCX)

S1 Prisma Checklist https://www.bmj.com/content/372/bmj.n71. (DOCX)
