# Supplementary material for: A systematic review and meta-analysis of incidence and spatiotemporal trends of snakebites in Iran
Source: PLoS Negl Trop Dis. 2025 Oct 14;19(10):e0013603. doi: 10.1371/journal.pntd.0013603 (PMC12520341; doi:10.1371/journal.pntd.0013603)
Supplement: S1 Table — (DOCX) [file pntd.0013603.s001.docx]

Table of reasons for exclusion of studies

| Row | Article deleted | Reason for article deletion |
| --- | --- | --- |
|  | Afroosheh M, Rastegar-Pouyani N, Rajabizadeh M, Kami HG. The brahminy blind snake, ramphotyphlops braminus (daudin, 1803), a newcomer to iran (ophidia: typhlopidae). Zoology in the Middle East. 2010;50(1):135-7. | Non-human study |
|  | Aghabiklooei A. The efficacy of aminophylline on raising consciousness in benzodiazepines-intoxicated patients. Asia Pacific Journal of Medical Toxicology. 2018;7(1):17-9. | Non-human study |
|  | Ahmadzadeh F, Carretero MA, Mebert K, Faghiri A, Ataei S, Hamid S, et al. Preliminary results on biological aspects of the grass snake, Natrix natrix in the southern coastal area of the Caspian Sea. Acta Herpetologica. 2011;6(2):209-21. | Non-human study |
|  | Akbarpour M, Rastegar-Pouyani N, Fathinia B, Rastegar-Pouyani E. A new species of the genus eirenis jan, 1863 (Squamata: Colubridae) from Kerman Province in South-central Iran. Zootaxa. 2020;4868(1):117-28. | Non-human study |
|  | Al Hatali BA, Al Mazroui SA, Alreesi AS, Geller RJ, Morgan BW, Kazzi ZN. Report of a Bite from a New Species of the Echis Genus--Echis omanensis. J Med Toxicol. 2015;11(2):242-4. | Non-human study |
|  | Alavi SM, Alavi L. Epidemiology of animal bites and stings in Khuzestan, Iran, 1997-2006. Journal of Infection and Public Health. 2008;1(1):51-5. | Lack of specific sample size  No reporting of incidence based on sample size |
|  | Alavi SM, Alavi L. Ten years survey of animal bites in Khuzestan. Pakistan Journal of Medical Sciences. 2009;25(3):370-4. | Lack of specific sample size  No reporting of incidence based on sample size |
|  | Alinejad S, Zamani N, Abdollahi M, Mehrpour O. A narrative review of acute adult poisoning in Iran. Iranian Journal of Medical Sciences. 2017;42(4):327-46. | The study is a review. |
|  | Allentoft ME, Rasmussen AR, Kristensen HV. Centuries-old DNA from an extinct population of aesculapian snake (zamenis longissimus) offers new phylogeographic insight. Diversity. 2018;10 | Non-human study |
|  | Allentoft ME, Rasmussen AR, Kristensen HV. Centuries-Old DNA from an Extinct Population of Aesculapian Snake (<i>Zamenis longissimus</i>) Offers New Phylogeographic Insight. Diversity-Basel. 2018;10(1). | Non-human study |
|  | Alshalah A, Williams DJ, Ferrario A. From fangs to antidotes: A scoping review on snakebite burden, species, and antivenoms in the Eastern Mediterranean Region. PLoS Negl Trop Dis. 2024;18(7):e0012200. | The study is a review. |
|  | Amanatfard E, Youssefi MR, Barimani A. Human Dermatitis Caused by Ophionyssus natricis, a Snake Mite. Iranian Journal of Parasitology. 2014;9(4):594-6. | The study is a case report. |
|  | Amini J. Road extraction from satellite images using a fuzzy-snake model. Cartographic Journal. 2009;46(2):164-72. | Non-human study |
|  | Amtaghri S, Akdad M, Slaoui M, Eddouks M. Traditional Uses, Pharmacological, and Phytochemical Studies of Euphorbia: A Review. Current Topics in Medicinal Chemistry. 2022;22(19):1553-70. | Non-human study |
|  | Amtaghri S, Eddouks M. Comprehensive review on the genus haloxylon: Pharmacological and phytochemical properties. Endocrine, Metabolic and Immune Disorders - Drug Targets. 2024;24(10):1146-60. | Non-human study |
|  | Ananjeva NB, Milto KD, Barabanov AV, Golynsky EA. An annotated type catalogue of amphibians and reptiles collected by Nikolay A. Zarudny in Iran and Middle Asia. Zootaxa. 2020;4722(2):101-28. | Non-human study |
|  | Ansari SH, Salehi S. Coagulopathy after spider bites in a six-year-old boy. Tehran University Medical Journal. 2008;65(12):84-7. | The study is a case report. |
|  | Asadi A, Salmanian A, Kaboli M. Eirenis thospitis schmidtler et lanza, 1990 (Reptilia: Colubridae): New to iran. Russian Journal of Herpetology. 2020;27(6):369-72. | Non-human study |
|  | Asgharian P, Delazar A, Asnaashari S. Chemical constituents of eremostachys macrophylla montbr. And Auch. aerial parts. Pharmaceutical Sciences. 2020;26(2):203-8. | Non-human study |
|  | Astaraki P, Basati G, Abbaszadeh S, Mahmoudi GA. A review of medicinal plants used for snakebites and scorpion stings in Iran: A systematic review. Research Journal of Pharmacy and Technology. 2020;13(3):1565-9. | The study is a review. |
|  | Astaraki P, Mahmoudi G, Ahadi M, Almasi V. The reasons of death among children and adolescents in Lorestan Province, west of Iran, from 2007 to 2014. International Journal of Medical Toxicology and Forensic Medicine. 2016;6(2):65-70. | Incidence rate not reported based on specific sample size |
|  | Asztalos M, Ayaz D, Bayrakci Y, Afsar M, Tok CV, Kindler C, et al. It takes two to tango - Phylogeography, taxonomy and hybridization in grass snakes and dice snakes (Serpentes: Natricidae: Natrix natrix, N. tessellata). Vertebrate Zoology. 2021;71:813-34. | Non-human study |
|  | Avci A, Gvoždík V, Jandzik D. Incongruence between taxonomy and genetics: Three divergent lineages within two subspecies of the rare Transcaucasian rat snake (Zamenis hohenackeri). Amphibia Reptilia. 2013;34(4):579-84. | Non-human study |
|  | Ayvazyan N, Mohamadvarzi M, Kirakosyan G, Ghulikyan L, Zaqaryan N. Morphological and functional alteration of human erythrocytes caused by some Iranian vipers' venom: Novel glance at the old problem. Acta Physiologica. 2016;217:42. | Non-human study |
|  | Babaie M, Salmanizadeh H, Zolfagharian H. Blood coagulation induced by Iranian saw-scaled viper (Echis carinatus) venom: Identification, purification and characterization of a prothrombin activator. Iranian Journal of Basic Medical Sciences. 2013;16(11):1145-50. | Non-human study |
|  | Bagci E, Aydin E, Mihasan M, Maniu C, Hritcu L. Anxiolytic and antidepressant-like effects of Ferulago angulata essential oil in the scopolamine rat model of Alzheimer's disease. Flavour and Fragrance Journal. 2016;31(1):70-80. | Non-human study |
|  | Barfaraz A, Harvey AL. The use of the chick biventer cervicis preparation to assess the protective activity of six international reference antivenoms on the neuromuscular effects of snake venoms in vitro. Toxicon. 1994;32(3):267-72. | Non-human study |
|  | Bastani B. A clinical review of snake bite with emphasis on species in Fars Province. Iranian Journal of Medical Sciences. 1979;10(1-4):163-200. | The study is a review. |
|  | Bazi A, Ghasempouri SK, Sahebnasagh A, Saghafi F. Snakebites by the sheltopusik (Pseudopus apodus) locally called Petilus snake to humans; a case report. Asia Pacific Journal of Medical Toxicology. 2019;8(3):104-6. | Non-human study |
|  | Blanco-Lapaz A, Mata-González M, Starkovich BM, Zeidi M, Conard NJ. Late Pleistocene environments in the southern Zagros of Iran and their implications for human evolution. Archaeological and Anthropological Sciences. 2022;14(8). | Non-human study |
|  | Carranza S, Arnold EN, Pleguezuelos JM. Phylogeny, biogeography, and evolution of two Mediterranean snakes, Malpolon monspessulanus and Hemorrhois hippocrepis (Squamata, Colubridae), using mtDNA sequences. Molecular Phylogenetics and Evolution. 2006;40(2):532-46. | Non-human study |
|  | Chafi MH, Eslamnezhad-Namin M, Dastjerdeh MS, Zareinejad MR, Oghalaie A, Bagheri KP, et al. CTXP, The Major Cobra Toxin Peptide from Naja Naja Oxiana Venom; A Promising Target for Antivenom Agent Development. Current Protein and Peptide Science. 2024;25(6):469-79. | Non-human study |
|  | Chefaoui RM, Hosseinzadeh MS, Mashayekhi M, Safaei-Mahroo B, Kazemi SM. Identifying suitable habitats and current conservation status of a rare and elusive reptile in Iran. Amphibia Reptilia. 2018;39(3):335-62. | Non-human study |
|  | Chen ZN, Shi SC, Vogel G, Ding L, Shi JS. Multiple lines of evidence reveal a new species of Krait (Squamata, Elapidae, Bungarus) from Southwestern China and Northern Myanmar. ZooKeys. 2021;2021(1025):35-71. | Non-human study |
|  | Dadar M, Nasiri V, Alamian S, Jafari H. A molecular and bacteriological survey of Brucella spp. in wild venomous snake in Iran. European Journal of Wildlife Research. 2024;70(1). | Non-human study |
|  | Dastgheib N, Kateb F, Hatam G. Recognizing the Correspondence of Alchemy and Sacred Art in Iran by Studying Luster-Ware Mihrabs. Bagh-E Nazar. 2021;18(97):19-32. | Non-human study |
|  | Dastranji N, Shojaeiyan A, Falahati-Anbaran M. Assessment of genetic diversity on snake melon (Cucumis melo var. flexuosus) using ISSR markers in Iran. InV International Symposium on Cucurbits 1151 2015 Jun 22 (pp. 45-50) | Non-human study |
|  | Dehghani R, Behrooz Fathi B, Pouyani NR, Chaharbaghi N. A report on Platyceps ventromaculatus (Colubridae) from Fereydan County in Isfahan province Iran. Iranian Journal of Animal Biosystematics. 2022;18(1):1-7. | Non-human study |
|  | Dehghani R, Mehrpour O, Shahi MP, Jazayeri M, Karrari P, Keyler D, et al. Epidemiology of venomous and semi-venomous snakebites (Ophidia: Viperidae, Colubridae) in the Kashan city of the Isfahan province in Central Iran. Journal of Research in Medical Sciences. 2014;19(1):33-40. | Lack of specific sample size  No reporting of incidence based on sample size |
|  | Dehghani R, Mohammadi MJ, Mostafaii G, Gilasi H, Shahrisvand B, Charkhloo E, Hoseindoost G. The frequency of stable fly larvae in the process of producing compost from municipal waste. Fresenius Environ Bull. 2018 Jan 1;27:5323-8. | Non-human study |
|  | Dehghani R, Monzavi SM, Mehrpour O, Shirazi FM, Hassanian-Moghaddam H, Keyler DE, et al. Medically important snakes and snakebite envenoming in Iran. Toxicon. 2023;230. | The study is a review. |
|  | Dehghani R, Pouyani NR, Tajaddini S, Varzandeh M. Platyceps karelini a nonvenomous snake (Ophidia: Colubridae) from Kashan county (Isfahan–Iran). Journal of Emergency Practice and Trauma. 2022;8(2):162-5. | Non-human study |
|  | Dehghani R, Sedaghat MM, Sabahi Bidgoli M. Wound myiasis due to musca domestica (Diptera: Muscidae) in persian horned viper, pseudocerastes persicus (Squamata: Viperidae). Journal of Arthropod-Borne Diseases. 2012;6(1):86-9. | Non-human study |
|  | Dehghani R, Sharif MR, Moniri R, Sharif A, Kashani HH. The identification of bacterial flora in oral cavity of snakes. Comparative Clinical Pathology. 2016;25(2):279-83. | Non-human study |
|  | Dezfoulian R, Mebert K, Karami M, Kaboli M, Ahmadzadeh F. Habitat factors determining the distribution of the caucasian Agama, Laudakia caucasia,(Squamata: Agamidae) in the Sorkh-e-hesar national park, Tehran province, Iran. Journal of Natural History. 2012 Nov 1;46(43-44):2735-47. | Non-human study |
|  | Emam SJ, Nikzamir A. Evaluation of hematological and biochemical parameters in snakebite patients referred to Razi Hospital, Ahwaz, Iran. Pakistan Journal of Medical Sciences. 2008;24(5):712-8. | Lack of specific sample size  No reporting of incidence based on sample size |
|  | Rahman R, Faiz MA, Selim S, Rahman B, Basher A, Jones A, d'Este C, Hossain M, Islam Z, Ahmed H, Milton AH. Annual incidence of snake bite in rural Bangladesh. PLoS neglected tropical diseases. 2010 Oct 26;4(10):e860. | Lack of definition of population |
|  | Eskandari N, Shafiee M, Mesgar AA, Zorzi F, Vidale M. A Copper Statuette from South-Eastern Iran (3rd Millennium B.C.). Iran and the Caucasus. 2022;26(1):17-31. | Non-human study |
|  | Eskandarzadeh N, Darvish J, Rastegar-Pouyani E, Ghassemzadeh F. Reevaluation of the taxonomic status of sand boas of the genus Eryx (Daudin, 1803) (Serpentes: Boidae) in Northeastern Iran. Turkish Journal of Zoology. 2013;37(3):348-56. | Non-human study |
|  | Eskandarzadeh N, Rastegar-Pouyani N, Rastegar-Pouyani E, Todehdehghan F, Rajabizadeh M. Sexual dimorphism in the javelin sand boa, Eryx jaculus (Linnaeus, 1758) (Serpentes: Erycidae), from Western Iran. Current Herpetology. 2018;37(1):88-92. | Non-human study |
|  | Eskandarzadeh N, Rastegar-Pouyani N, Rastegar-Pouyani E, Todehdehghan F, Rajabizadeh M, Zarrintab M, Rhadi FA, Kami HG. Revised classification of the genus Eryx Daudin, 1803 (Serpentes: Erycidae) in Iran and neighbouring areas, based on mtDNA sequences and morphological data. Herpetological Journal. 2020 Jan 1;30(1). | Non-human study |
|  | Esmaili A, Kamyab M, Fatemikia H, Ahmadzadeh H, Movahed A, Kim E, et al. Experimental evaluation of mouse hind paw edema induced by iranian naja oxiana venom. Archives of Razi Institute. 2021;76(1):139-47. | Non-human study |
|  | Ettling JA, Aghasyan LA, Aghasyan AL, Parker PG. Spatial ecology of Armenian vipers, Montivipera raddei, in a human-modified landscape. Copeia. 2013(1):64-71. | Non-human study |
|  | Faghiri A, Shiravi A, Hojati V, Kami HG. Observations on the spermatogenic cycle of the Grass snake, Natrix natrix (Serpentes: Colubridae) in Northern Iran. Asian Herpetological Research. 2011;2(1):55-9. | Non-human study |
|  | Fallahi N, Shahbazzadeh D, Maleki F, Aghdasi M, Tabatabaie F, Khanaliha K. The in vitro study of anti-leishmanial effect of Naja naja oxiana snake venom on Leishmania major. Infectious Disorders - Drug Targets. 2020;20(6):913-9. | Non-human study |
|  | Farajidana H, Mosalamiaghili S, Assadian K, Jahangiri S, Masumzadegan M, Sadeghi F, et al. Treatment response and clinical features of snakebite envenomation in Alborz province, Iran: A cross-sectional study. Health Science Reports. 2024;7(10). | Lack of specific sample size  No reporting of incidence based on sample size |
|  | Farzad R, Gholami A, Hayati Roodbari N, Shahbazzadeh D. The anti-rabies activity of Caspian cobra venom. Toxicon. 2020;186:175-81. | Non-human study |
|  | Fatehi-Hassanabad Z, Fatehi M. Characterisation of some pharmacological effects of the venom from Vipera lebetina. Toxicon. 2004;43(4):385-91. | Non-human study |
|  | Fatemizadeh F, Mohammadi A, Kaboli M. Captive breeding and ex-situ conservation of the Caucasian pit viper Gloydius caucasicus. Herpetological Bulletin. 2023(165):5-10. | Non-human study |
|  | Fathi B, Younesi F, Salami F. Research Paper Acute Venom Toxicity Determinations for Five Iranian Vipers and a Scorpion. Iranian Journal of Toxicology. 2022;16(2):73-82. | Non-human study |
|  | Fathinia B, Rastegar-Pouyani E, Rastegar-Pouyani N, Darvishnia H. A new species of the genus Rhynchocalamus Günther, 1864 (Reptilia: Squamata: Colubridae) from Ilam province in western Iran. Zootaxa. 2017;4282(3):473-86. | Non-human study |
|  | Fathinia B, Rastegar-Pouyani E, Shafaeipour A. A new species of Eirenis (Ophidia: Colubridae) from highland habitats in southern Iran. Zoology in the Middle East. 2019;65(4):319-29. | Non-human study |
|  | Fathinia B, Rastegar‐Pouyani N, Rastegar‐Pouyani E. Molecular phylogeny and historical biogeography of genera Eristicophis and Pseudocerastes (Ophidia, Viperidae). Zoologica scripta. 2018 Nov;47(6):673-85. | Non-human study |
|  | Fathinia B, Rastegar-Pouyani N, Rastegar-Pouyani E, Todehdehghan F, Amiri F. Avian deception using an elaborate caudal lure in Pseudocerastes urarachnoides (Serpentes: Viperidae). Amphibia-Reptilia. 2015 Jan 1;36(3):223-31. | Non-human study |
|  | Fathinia B, Rastegar-Pouyani N, Rastegar-Pouyani E, Todehdehghan F, Mansouri M. Annual activity pattern of Pseudocerastes urarachnoides Bostanchi, Anderson, Kami & Papenfuss, 2006, with notes on its natural history (squamata: Serpentes: Viperidae). Herpetozoa. 2017 Jan 30;29(3-4):135-42. | Non-human study |
|  | Feyzabadi VY, Mohammadi NK, Omidvar N, Karimi-Shahanjarini A, Nedjat S, Rashidian A. Factors Associated With Unhealthy Snacks Consumption Among Adolescents in Iran's Schools. International Journal of Health Policy and Management. 2017;6(9):519-28. | Lack of specific sample size  No reporting of incidence based on sample size |
|  | Firoozfar F, Saghafipour A, Jesri N. Scorpions and Their Human Mortality Report in Iran: A Review Article. Iranian Journal of Public Health. 2019;48(12):2140-53. | The study is a review. |
|  | Flood FB. Image against nature: Spolia as apotropaia in Byzantium and the dār al-Islām. Medieval History Journal. 2006;9(1):143-66. | Non-human study |
|  | Fritz U, Schmidtler JF. The Fifth Labour of Heracles: Cleaning the Linnean stable of names for grass snakes (Natrix astreptophora, N. helvetica, N. natrix sensu stricto). Vertebrate Zoology. 2020;7(4):661-5. | Non-human study |
|  | Ganjali M, Keighobadi M, Khedri J. First report of Ophidascaris filaria (Nematode: Ascarididae) from the Indian python in Sistan, Iran. Comparative Clinical Pathology. 2015;24(5):1285-8. | Non-human study |
|  | Gharzi A, Yari A. Age determination in the snake-eyed lizard, ophisops elegans, by means of skeletochronology (Reptilia: Lacertidae). Zoology in the Middle East. 2013;59(1):10-5. | Non-human study |
|  | Ghezellou P, Albuquerque W, Garikapati V, Casewell NR, Kazemi SM, Ghassempour A, et al. Integrating Top-Down and Bottom-Up Mass Spectrometric Strategies for Proteomic Profiling of Iranian Saw-Scaled Viper, Echis carinatus sochureki, Venom. Journal of Proteome Research. 2021;20(1):895-908. | Non-human study |
|  | Ghezellou P, Dillenberger M, Kazemi SM, Jestrzemski D, Hellmann B, Spengler B. Comparative Venom Proteomics of Iranian, Macrovipera lebetina cernovi, and Cypriot, Macrovipera lebetina lebetina, Giant Vipers. Toxins. 2022;14(10). | Non-human study |
|  | Gholamifard A, Şahin MK. Range dynamics of Walterinnesia morgani (Serpentes, Elapidae) during climatic oscillations in Iran. Herpetozoa. 2023;36:317-24. | Non-human study |
|  | Ghorbanpur M, Zare Mirakabadi A, Zokaee F, Zolfagarrian H, Rabiei H. Purification and partial characterization of a coagulant serine protease from the venom of the Iranian snake Agkistrodon halys. Journal of Venomous Animals and Toxins including Tropical Diseases. 2009;15:411-23. | Non-human study |
|  | Ghorbanpur M, Zare Mirakabadi A, Zokaee F, Zolfagarrian H. Identification and partial purification of an anticoagulant factor from the venom of the Iranian snake Agkistrodon halys. Journal of Venomous Animals and Toxins Including Tropical Diseases. 2010;16(1):96-106. | Non-human study |
|  | Ghorbanpur M, Zare Mirakabadi A, Zokaee F, Zolfagarrian H, Rabiei H. Purification and partial characterization of a coagulant serine protease from the venom of the Iranian snake Agkistrodon halys. Journal of Venomous Animals and Toxins Including Tropical Diseases. 2009;15(3):411-23. | Non-human study |
|  | Ghukasyan G, Mohamadvarzi M, Kirakosyan G, Ghulikyan L, Ayvazyan N. Morphological and functional alteration of human erythrocytes caused by some Iranian vipers' venom. Toxicon. 2019;159:S31. | Non-human study |
|  | Gorelik A, Tsybriy A, Tsybriy V. What did the Skull of Aurochs, an axe and female statuettes tell us abouti (on the problem of the initial neolithisation on the Lower Don). Stratum Plus. 2014;2014(2):247-82. | Non-human study |
|  | Goudarzi HR, Salehi Najafabadi Z, Movahedi A, Noofeli M. Bradykinin-potentiating factors of venom from iranian medically important scorpions. Archives of Razi Institute. 2019;74(4):385-94. | Non-human study |
|  | GRĂDINARU E, Gaetani M. Upper Spathian to Bithynian (Lower to Middle Triassic) brachiopods from North Dobrogea (Romania). Rivista italiana di Paleontologia e Stratigrafia. 2019 Oct;125(1). | Non-human study |
|  | Guicking D, Joger U, Wink M. Cryptic diversity in a Eurasian water snake (Natrix tessellata, Serpentes: Colubridae): Evidence from mitochondrial sequence data and nuclear ISSR-PCR fingerprinting. Organisms Diversity and Evolution. 2009;9(3):201-14. | Non-human study |
|  | Halajian A, Bursey CR, Goldberg SR, Gol SMA. Helminth parasites of the european glass lizard, pseudopus apodus (Squamata: Anguidae), and European grass snake, natrix natrix (Serpentes: Colubridae), from Iran. Comparative Parasitology. 2013;80(1):151-6. | Non-human study |
|  | Harvey AL, Barfaraz A, Thomson E, Faiz A, Preston S, Harris JB. Screening of snake venoms for neurotoxic and myotoxic effects using simple in vitro preparations from rodents and chicks. Toxicon. 1994;32(3):257-65. | Non-human study |
|  | Hassanian-Moghaddam H, Monzavi SM, Shirazi FM, Warrell DA, Mehrpour O. First report of a confirmed case of Montivipera latifii (Latifi's viper) envenoming and a literature review of envenoming by Montivipera species. Toxicon. 2022;207:48-51. | Non-human study |
|  | Heidarpour M, Ennaifer E, Ahari H, Srairi-Abid N, Borchani L, Khalili G, Amini H, Anvar AA, Boubaker S, El-Ayeb M, Shahbazzadeh D. Histopathological changes induced by Hemiscorpius lepturus scorpion venom in mice. Toxicon. 2012 Mar 1;59(3):373-8. | Non-human study |
|  | Heydari Sereshk Z, Riyahi Bakhtiari A. Distribution patterns of PAHs in different tissues of annulated sea snake (Hydrophis cyanocinctus) and short sea snake (Lapemis curtus) from the Hara Protected Area on the North Coast of the Persian Gulf, Iran. Ecotoxicology and Environmental Safety. 2014;109:116-23. | Non-human study |
|  | Hojati V, Faghiri A, Shiravi A. Diet of the grass snake, Natrix natrix (Linnaeus, 1758)(Serpentes: Colubridae), in northern Iran. Zoology in the Middle East. 2012 Jan 1;55(1):132-4. | Non-human study |
|  | Yousefkhani SS, Mirshamsi O, Ilgaz C, Kumlutaş Y, Avci A. Ecological niche divergence between Trapelus ruderatus (Olivier, 1807) and T. persicus (Blanford, 1881)(Sauria: Agamidae) in the Middle East. Asiatic Herpetol. Res. 2016;7(2):96-102. | Non-human study |
|  | Hosseinzadeh MS, Qomi MF, Naimi B, Roedder D, Kazemi SM. Habitat suitability and modelling the potential distribution of the Plateau Snake Skink Ophiomorus nuchalis (Sauria Scincidae) on the Iranian Plateau. North-western journal of zoology. 2018 Jun;14(1):60-3. | Non-human study |
|  | Hosseinzadeh MS, Ghezellou P, Kazemi SM. Predicting the potential distribution of the endemic snake Spalerosophis microlepis (Serpentes: Colubridae), in the Zagros mountains, western Iran. Salamandra. 2017;53(2):294-8. | Non-human study |
|  | Hosseinzadeh MS, Farhadi Qomi M, Kazemi SM. Distribution of Ophiomorus nuchalis Nilson & Andrén, 1978: Current status of knowledge. Herpetozoa. 2016 Jul 30;29(1-2):92-5. | Non-human study |
|  | Hritcu L, Bagci E, Aydin E, Mihasan M. Antiamnesic and Antioxidants Effects of Ferulago angulata Essential Oil Against Scopolamine-Induced Memory Impairment in Laboratory Rats. Neurochemical Research. 2015;40(9):1799-809. | Non-human study |
|  | Hussain S, Bukhari SM, Rehman KU, Javid A, Hussain J. First Record of Eryx sistanensis (Squamata, Boidae) from Pakistan and Evaluation of Potential Threats of the Species. Russian Journal of Herpetology. 2024 Dec 21;31(6):326-36. | Non-human study |
|  | Ismail M, Memish ZA. Venomous snakes of Saudi Arabia and the Middle East: a keynote for travellers. Int J Antimicrob Agents. 2003;21(2):164-9. | Non-human study |
|  | Jablonski D, Ahmed SH. The first record of the rarely observed rat snake, Elaphe urartica Jablonski et al., 2019 (Squamata, Colubridae) for Iraq. Herpetozoa. 2024;37:73-6. | Non-human study |
|  | Jablonski D, Koleska D. Molecular identification of Eremias stummeri (Squamata: Lacertidae) as a prey for Gloydius halys complex (Serpentes: Viperidae) from Kyrgyzstan. Phyllomedusa. 2017;16(1):121-4. | Non-human study |
|  | Jablonski D, Nagy ZT, Avci A, Olgun K, Kukushkin OV, Safaei-Mahroo B, et al. Cryptic diversity in the smooth snake (Coronella austriaca). Amphibia Reptilia. 2019;40(2):179-92. | Non-human study |
|  | Jafari H, Frhangpazhouh F, Kharazi P, Moghadam ST, Salabi F, Forouzan A. Identification and Determination of Epidermal Fatty Acids Extracted in Walterinnesia morgani (Elapidae) Using GC/FID. Proceedings of the Zoological Society. 2020;73(4):418-21. | Non-human study |
|  | Jalali A, Mohajer S, Iran ZJ, Kazemi SM. The need to expand cover of polyvalent snake antivenom in Iran. Toxicon. 2024;238. | Non-human study |
|  | Jameie F, Nasiri V, Paykari H. Morphological detection and molecular characterization of Hepatozoon spp. from venomous terrestrial snakes in Iran. Experimental Parasitology. 2022;239. | Non-human study |
|  | Jandzik D, Avcı A, Gvoždík V. Incongruence between taxonomy and genetics: three divergent lineages within two subspecies of the rare Transcaucasian rat snake (Zamenis hohenackeri). Amphibia-Reptilia. 2013 Jan 1;34(4):579-84. | Non-human study |
|  | Jridi I, Catacchio I, Majdoub H, Shahbazeddah D, El Ayeb M, Frassanito MA, et al. Hemilipin, a novel Hemiscorpius lepturus venom heterodimeric phospholipase A2, which inhibits angiogenesis in vitro and in vivo. Toxicon. 2015;105:34-44. | Non-human study |
|  | Jridi I, Catacchio I, Majdoub H, Shahbazeddah D, El Ayeb M, Frassanito MA, Ribatti D, Vacca A, Borchani L. Hemilipin, a novel Hemiscorpius lepturus venom heterodimeric phospholipase A2, which inhibits angiogenesis in vitro and in vivo. Toxicon. 2015 Oct 1;105:34-44. | Non-human study |
|  | Kadkhodazadeh M, Rajabibazl M, Motedayen M, Shahidi S, Malekshahi ZV, Rahimpour A, et al. Isolation of polyclonal single-chain fragment variable (Scfv) antibodies against venomous snakes of iran and evaluation of their capability in neutralizing the venom. Iranian Journal of Pharmaceutical Research. 2020;19(3):288-96. | Non-human study |
|  | Kamiguti AS, Theakston RDG, Tomy SC. An investigation of the coagulant activity of the venom of the saw-scaled viper (Echis carinatus) from Saudi Arabia. Annals of Tropical Medicine and Parasitology. 1988;82(5):503-9. | Non-human study |
|  | Kamyab M, Kim E, Hoseiny SM, Seyedian R. Enzymatic analysis of iranian echis carinatus venom using zymography. Iranian Journal of Pharmaceutical Research. 2017;16(3):1157-62. | Non-human study |
|  | Karamiani R, Hosseini M. Modeling the past and contemporary habitat suitability and distribution of the Levantine viper Macrovipera lebetinus (Linnaeus, 1758) (Ophidia: Viperidae). Journal of Wildlife and Biodiversity. 2023;8(1):39-53. | Non-human study |
|  | Karamiani R, Rastegar-Pouyani N, Rastegar-Pouyani E. Sexual dimorphism in the asian snake-eyed skink, Ablepharus pannonicus (Fitzinger, 1823 (sauria): Scincidae) from Iran. Russian Journal of Herpetology. 2018;25(1):1-5. | Non-human study |
|  | Karamiani R, Rastegar-Pouyani N, Rastegarpouyani E. Modeling the past and current distribution and habitat suitability for Ablepharus grayanus and A. pannonicus (Sauria: Scincidae). Asian herpetological research. 2018;9(1):56-64A. | Non-human study |
|  | Karamiani R, Rastegar-Pouyani N, Rastegar-Pouyani E. Systematics and distribution of the genus Ablepharus Fitzinger, 1823 (Sauria, Scincidae): A review. Iranian Journal of Animal Biosystematics. 2021;17(2):115-29. | Non-human study |
|  | Karamiani R, Rastegar-Pouyani N, Rastegar-Pouyani E. Phylogenetic relationships amongst the snake-eyed lizards of the genus Ablepharus Fitzinger, 1823 (Sauria, Scincidae) in the Iranian Plateau based on mtDNA sequences. Herpetozoa. 2021;34:183-94. | Non-human study |
|  | Karamiani R, Rastegar-Pouyani N, Rastegar-Pouyani E, Akbarpour M, Damadi E. Verification of the Minor Snake-eyed Skink, Ablepharus grayanus (Stoliczka, 1872) (Sauria: Scincidae), from Iran. Zoology in the Middle East. 2015;61(3):226-30. | Non-human study |
|  | Kashkooli S, Khamehchian S, Dabaghian M, Namvarpour M, Tebianian M. Effects of Adjuvant and Immunization Route on Antibody Responses against Naja Naja oxiana Venom. Archives of Razi Institute. 2023;78(5):1177-83. | Non-human study |
|  | Kazemi SM, Al-Sabi A, Long C, Shoulkamy MI, Abd El-Aziz TM. Case Report: Recent Case Reports of Levant Blunt-Nosed Viper Macrovipera lebetina obtusa Snakebites in Iran. Am J Trop Med Hyg. 2021;104(5):1870-6. | The study is a case report. |
|  | Kazemi SM, Hosseinzadeh MS, Weinstein SA. Identifying the geographic distribution pattern of venomous snakes and regions of high snakebite risk in Iran. Toxicon. 2023;231. | Lack of definition of population |
|  | Kazemi SM, Jahan-Mahin MH, Mohammadian-Kalat T, Hosseinzadeh MS, Weinstein SA. Local envenoming by the coinsnake or Asian racer, Hemorrhois nummifer and mountain racer or leopard snake, Hemorrhois ravergieri (Serpentes: Colubridae, Colubrinae) in Iran: A reminder of the importance of species identification in the medical management of snakebites. Toxicon. 2023;226:107070. | Non-human study |
|  | Kazemi SM, Jahan-Mahin MH, Zangi B, Khozani RS, Warrell DA. A case of envenoming by a Persian false-horned viper Pseudocerastes persicus (Duméril, Bibron & Duméril, 1854) (Serpentes: Viperidae) in Southeastern Iran. Toxicon. 2023;223:107009. | Non-human study |
|  | Kazemi SM, Kelisani ZG, Avella I, Luddecke T. The need for a refined scorpion antivenom for Iran. Toxicon. 2024;248. | Non-human study |
|  | Khani S, Kami HG, Rajabizadeh M. Geographic variation of Gloydius halys caucasicus (Serpentes: Viperidae) in Iran. Zoology in the Middle East. 2017 Oct 2;63(4):303-10. | Non-human study |
|  | Khoobdel M, Jafari H, Akhoond MR. The impacts of the introduced Indian gray mongoose Herpestes edwardsii (Mammalia, Carnivora) on the non-target native species of Abu-Musa Island, Iran. Journal of Military Medicine. 2016;18(1):371-9. | Non-human study |
|  | Khoobdel M, Nikbakhtboroujeni G, Zahraeisalehi T, Khosravi M, Sasani F, Bokaei S, et al. Diagnosis of Mesobuthus eupeus envenomation by skin test: Reverse passive Arthus reaction. Toxicon. 2014;77:133-40. | Non-human study |
|  | Kidov AA. Rediscovery of the Persian dwarf snake (Eirenis persicus, Ophidia, Colubridae) in Qazvin Province, Iran. Зоологический журнал. 2021;100(6):690-2. | Non-human study |
|  | Kidov AA, Kondratova TE. Morphometric and Reproductive Features of the Two-Streaked Snake-Eyed Skink (Ablepharus bivittatus (Menetries 1832), Reptilia, Scincidae) in the Talysh Mountains. Biology Bulletin. 2021;48(9):1487-93. | Non-human study |
|  | Kidov AA, Kondratova TE, Ivolga RA, Lyapkov SM. Age Structure, Growth, and Reproduction of the Twin-Striped Skink (Ablepharus bivittatus, Reptilia, Scincidae) in the Talysh Mountains (Ardabil Province, Iran). Biology Bulletin. 2023 Dec;50(9):2271-7. | Non-human study |
|  | Kirakosyan G, Mohamadvarzi M, Ghulikyan L, Zaqaryan N, Kishmiryan A, Ayvazyan N. Morphological and functional alteration of erythrocyte ghosts and giant unilamellar vesicles caused by Vipera latifi venom. Comparative Biochemistry and Physiology Part - C: Toxicology and Pharmacology. 2016;190:48-53. | Non-human study |
|  | Krysa-Clark J, Lewis S, Waterworth TA. Management of a snake bite in the field. J R Army Med Corps. 2004;150(2):97-8. | Non-human study |
|  | Lak R, Hajari A, Beni MN. Accidents in children under 5 years in Isfahan, Iran. Iranian Journal of Pediatrics. 2014;24(3):336. | Lack of specific sample size  No reporting of incidence based on sample size |
|  | Larki S, Alborzi A, Bahramnejad K, Asadi Z. The Occurrence of the Strongylid Nematodes, Kalicephalus viparae viparae (Nematoda: Diaphanocephalidae), in Viper Snakes, Macrovipera lebetina (Reptilia: Viperidae), Southwestern Iran. Archives of Razi Institute. 2023;78(2):611-8. | Non-human study |
|  | Larypoor M, Mohammad Hassan Z, Yadegari MH, Akhavan Sepahy A. Evaluation of the susceptibility of dermatophytes to garlic extract. Yakhteh. 2006;8(29):7-16+66. | Non-human study |
|  | Latifi M. Variation in yield and lethality of venoms from Iranian snakes. Toxicon. 1984;22(3):373-80. | Non-human study |
|  | Latifi M, Tabatabai M. Immunological studies on Iranian scorpion venom and antiserum. Toxicon. 1979;17(6):617-20. | Non-human study |
|  | Lee JL, Yushchenko PV, Milto KD, Rajabizadeh M, Pouyani ER, Jablonski D, et al. Kukri snakes Oligodon Fitzinger, 1826 of the Western Palearctic with the resurrection of Contia transcaspica Nikolsky, 1902 (Reptilia, Squamata, Colubridae). PeerJ. 2023;11. | Non-human study |
|  | Louei Monfared A. Microscopic study on characterization of peripheral blood cells of Levantine viper (Macrovipera lebetina obtusa) from southwestern Iran. Comparative Clinical Pathology. 2014;23(3):755-9. | Non-human study |
|  | Mahmoudi GA, Ahadi M, Fouladvand A, Rezaei B, Bodagh Z, Astaraki P. Evaluation of Allergic Reactions Following Intravenous Infusion of Polyvalent Antivenom in Snakebite Patients. Anti-Inflammatory and Anti-Allergy Agents in Medicinal Chemistry. 2021;20(4):367-72. | Non-human study |
|  | Mashhadi I, Kavousi Z, Peymani P, Salman Zadeh Ramhormozi S, Keshavarz K. Economic burden of scorpion sting and snake bite from a social perspective in Iran. Shiraz E Medical Journal. 2017;18(8). | Lack of specific sample size  No reporting of incidence based on sample size |
|  | Mashkour M, Monchot H, Trinkaus E, Reyss JL, Biglari F, Bailon S, et al. Carnivores and their prey in the Wezmeh cave (Kermanshah, Iran): A late pleistocene refuge in the zagros. International Journal of Osteoarchaeology. 2009;19(6):678-94. | Non-human study |
|  | Mendoza Forrest SK, Skjærvø PO. Witches, whores, and sorcerers: The concept of evil in early Iran2011. 1-231 p. | Non-human study |
|  | Mirzajani A, Naderi S, Ganeh A, Hadipour E, Salahi M, Javidpour J. Trophic flexibility of Eurasian otter (Lutra lutra) in Anzali Wetland, Iran, assessed by fecal and stable isotope analysis. Aquatic Ecology. 2021;55(2):401-15. | Non-human study |
|  | Moadab M, Zargan J, Rastegar-Pouyani E, Hajinourmohammadi A. Modelling the potential distribution of spalerosophis diadema (Schlegel, 1837) (Serpents: Colubridae) in Iran. Herpetology Notes. 2018;11:805-8. | Non-human study |
|  | Mohamed FH, Abo-Zeid AAI, Abd El-Hamed KE, Elwan MW, Salam MMA. Genetic Diversity in Egyptian Snake Melon Accessions as Revealed by Inter Simple Sequence Repeat (ISSR) Markers. Catrina-the International Journal of Environmental Sciences. 2020;22(1):71-6. | Non-human study |
|  | Moini M, Peyvandi AA, Rasouli MR, Khajei A, Kakavand M, Eghbal P, et al. Pattern of animal-related injuries in Iran. Acta Medica Iranica. 2011;49(3):163-8. | Lack of specific sample size  No reporting of incidence based on sample size |
|  | Mondal S, Ganesh SR, Raghunathan C. Some rare species of sea snakes (Squamata: Serpentes: Elapidae: Hydrophiinae: Hydrophis, Microcephalophis) from the Indian Coasts and nearby waters, lodged in major systematic Indian zoological collections. Bonn Zoological Bulletin. 2023;72(2):209-22. | Non-human study |
|  | Monzavi SM, Afshari R, Khoshdel AR, Mahmoudi M, Salarian AA, Samieimanesh F, et al. Analysis of effectiveness of Iranian snake antivenom on Viper venom induced effects including analysis of immunologic biomarkers in the Echis carinatus sochureki envenomed victims. Toxicon. 2019;158:38-46. | Non-human study |
|  | Morad R, Zohreh A, Mozhgan N. Study of alkaline phosphatase activity in isolation fractions from Iranian snake Vipera lebetina venom. Clinical Biochemistry. 2011 Sep 1;44(13):S88-9. | Non-human study |
|  | Moradi N, Joger U, Bafti SS, Sharifi A, SehhatiSabet ME. Biogeography of the Iranian snakes. PLoS ONE. 2024;19(10 October). | Lack of definition of population |
|  | Motedayen MH, Nikbakht Brojeni GH, Rasaee MJ, Zare Mirakabadi A, Khorasani A, Eizadi H, et al. Production of a human recombinant polyclonal fab antivenom against iranian viper echis carinatus. Archives of Razi Institute. 2018;73(4):287-94. | Non-human study |
|  | Nasiri V, Jameie F. Snake neosporosis: molecular detection and phylogenic characterization of Neospora caninum DNA from Iranian venomous snakes. European Journal of Wildlife Research. 2023;69(4). | Non-human study |
|  | Sagheb MM, Sharifian M, Moini M, Salehi O. Clinical features of snake bite in southern Iran. Tropical Doctor. 2011;41(4):236-7. | Lack of access to full text |
|  | Senthilkumaran S, Rizwan T, Elangovan N, Usman MS, Menezes RG, Thirumalaikolundusubramanian P. Visual Hallucinations After a Russell's Viper Bite. Wilderness & Environmental Medicine. 2021;32(3):351-4. | Non-human study |
|  | Shahbaz A, Allahverdi N, Parizad N. "It's like the snakes and ladders game.''; lived experience of patients with multiple sclerosis regarding their return to work: A qualitative study. Current Psychology. 2023;42(21):18255-67. | Non-human study |
|  | Shahrajabian MH, Sun WL. Iranian Traditional Medicine (ITM) and Natural Remedies for Treatment of the Common Cold and Flu. Reviews on Recent Clinical Trials. 2024;19(2):91-100 | Non-human study |
|  | Torki F. A new species of blind snake, Xerotyphlops, from Iran. Herpetological Bulletin. 2017(140):1-5. | Non-human study |
|  | Yousefi M, Yousefkhani SH, Grünig M, Kafash A, Rajabizadeh M, Pouyani ER. Identifying high snakebite risk area under climate change for community education and antivenom distribution. Sci Rep. 2023;13(1):8191. | Non-human study |
|  | Yousefkhani SSH, Yousefi M, Khani A, Pouyani ER. Snake fauna of Shirahmad Wildlife Refuge and Parvand protected area, Khorasan Razavi province, Iran. Herpetology Notes. 2014;7(0):75-82. | Non-human study |
|  | Rezaie-Atagholipour M, Riyahi-Bakhtiari A, Rajabizadeh M, Ghezellou P. Status of the annulated sea snake, hydrophis cyanocinctus, in the hara protected area of the persian gulf: (Reptilia: Elaphidae: Hydrophiinae). Zoology in the Middle East. 2012;57(1):53-60. | Non-human study |
|  | Sadeghi N, Rastegar-Pouyani N, Yousefkhani SSH. Sexual size dimorphism in Eirenis collaris Ménétriés 1832 from Iran. Russian Journal of Herpetology. 2014;21(4):291-4. | Non-human study |
|  | Safaei-Mahroo B, Ghaffari H, Fahimi H, Broomand S, Yazdanian M, Najafi Majd E, et al. The Herpetofauna of Iran: Checklist of Taxonomy, Distribution and Conservation Status. Asian Herpetological Research. 2015;6(4):257-90. | Non-human study |
|  | Safdarian P, Todehdehghanb F, Hojatia V, Shiravi A. Seasonal changes in the testicular activity of the Iranian Mountain Viper, Montivipera albicornuta (Nilson & Andrén, 1985) (Reptilia: Viperidae). Zoology in the Middle East. 2016;62(1):39-45. | Non-human study |
|  | Sagheb MM, Sharifian M, Moini M, Salehi O. Acute renal failure and acute necrotizing pancreatitis after Echis carinatus sochureki bite, report of a rare complication from southern Iran. Prague medical report. 2011;112(1):67-71. | Non-human study |
|  | Krysa-Clark J, Lewis S, Waterworth TA. Management of a snake bite in the field. Journal of the Royal Army Medical Corps. 2004;150(2):97-8. | Non-human study |
|  | Lak R, Hajari A, Beni MN. Accidents in children under 5 years in Isfahan, Iran. Iranian Journal of Pediatrics. 2014;24(3):336. | Lack of specific sample size  No reporting of incidence based on sample size |
|  | Licata F, Pola L, Šmíd J, Ibrahim AA, Liz AV, Santos B, et al. The missing piece of the puzzle: A new and widespread species of the genus Rhynchocalamus Günther, 1864 (Squamata, Colubridae) from the Arabian Peninsula. Zoosystematics and Evolution. 2024;100(2):691-704. | Non-human study |
|  | Dehghani R, Mehrpour O, Shahi MP, Jazayeri M, Karrari P, Keyler D, et al. Epidemiology of venomous and semi-venomous snakebites (Ophidia: Viperidae, Colubridae) in the Kashan city of the Isfahan province in Central Iran. Journal of Research in Medical Sciences. 2014;19(1):33-40. | Lack of specific sample size  No reporting of incidence based on sample size |
|  | Hafezi G, Rahmani AH, Soleymani M, Nazari P. An epidemiologic and clinical study of snake bites during a five-year period in Karoon, Iran. Asia Pacific Journal of Medical Toxicology. 2018;7(1):13-6. | Lack of specific sample size  No reporting of incidence based on sample size |
|  | Kassiri H, Naghibzadeh R, Kavosi-Asl P. An epidemiological study of patients with snake biting in the health centre of Bandar Mahshahr, SW Iran. Asian Journal of Animal and Veterinary Advances. 2012;7(3):268-72. | Lack of specific sample size  No reporting of incidence based on sample size |
|  | Afroz A, Siddiquea BN, Chowdhury HA, Jackson TN, Watt AD. Snakebite envenoming: A systematic review and meta-analysis of global morbidity and mortality. PLoS neglected tropical diseases. 2024;18(4):e0012080. | The study is a review. |
|  | Chippaux J-P. Snakebite envenomation turns again into a neglected tropical disease! Journal of venomous animals and toxins including tropical diseases. 2017;23:38. | Non-human study |
|  | Rahmani AH, Badiee MS, Vadizadeh A, Godarzi H. The Investigation of the Clinical Symptoms of Snakebites in Children Admitted to Abuzar Hospital in South of Iran from 2018-2020. Asia Pacific Journal of Medical Toxicology. 2023;12(1):29-31. | Lack of specific sample size  No reporting of incidence based on sample size |
|  | Sagheb MM, Sharifian M, Moini M, Salehi O. Clinical features of snake bite in southern Iran. Tropical doctor. 2011;41(4):236-7. | Lack of specific sample size  No reporting of incidence based on sample size |
|  | Sattar MN. Identification and molecular analysis of watermelon chlorotic stunt virus infecting snake gourd in Saudi Arabia. Notulae Botanicae Horti Agrobotanici Cluj-Napoca. 2024;52(3). | Non-human study |
|  | Savasari RB, Shiravi A, Hojati V. The male reproductive cycle of the Dice Snake, Natrix tessellata (Serpentes: Colubridae), in northern Iran. Zoology in the Middle East. 2015;61(1):18-25. | Non-human study |
|  | Schwartz M. Transformations of the Indo-Iranian Snake-man: Myth, Language, Ethnoarcheology, and Iranian Identity. Iranian Studies. 2012;45(2):275-9. | Non-human study |
|  | Senthilkumaran S, Rizwan T, Elangovan N, Usman MS, Menezes RG, Thirumalaikolundusubramanian P. Visual Hallucinations After a Russell's Viper Bite. Wilderness & Environmental Medicine. 2021;32(3):351-4. | Non-human study |
|  | Sepaskhah AR, Shaabani MK. Infiltration and hydraulic behaviour of an anguiform furrow in heavy texture soils of Iran. Biosystems Engineering. 2007;98(2):248-56. | Non-human study |
|  | Sepehri G, Heidari MR, Tezerji RS. The effects of bezoar on the Echis carinatus snake venom poisoning in mice. Iranian Journal of Pharmacology and Therapeutics. 2007;6(2):203-6. | Non-human study |
|  | Sereshk ZH, Bakhtiari AR. Distribution patterns of PAHs in different tissues of annulated sea snake (Hydrophis cyanocinctus) and short sea snake (Lapemis curtus) from the Hara Protected Area on the North Coast of the Persian Gulf, Iran. Ecotoxicology and environmental safety. 2014 Nov 1;109:116-23. | Non-human study |
|  | Dehghani R, Rastegar Pouyani N, Dadpour B, Keyler D, Panjehshahi M, Jazayeri M, et al. A survey on non-venomous snakes in Kashan (Central Iran). Journal of Biology and Today's World. 2016;5(4):65-75. | Lack of access to full text |
|  | Shafaeipour A. Breeding long-legged Buzzard Buteo rufinus in forests of southwestern Iran: Feeding habits and reproductive performance. Turkish Journal of Zoology. 2015;39(4):702-7. | Non-human study |
|  | Shafiei S, Fahimi H, Sehhatisabet ME, Moradi N. Rediscovery of Maynard's Longnose sand snake, Lytorhynchus maynardi, with the geographic distribution of the genus Lytorhynchus Peters, 1863 in Iran. Zoology in the Middle East. 2015;61(1):32-7. | Non-human study |
|  | Shah AA, Badshah L, Muhammad M, Khan S, Abbas M, Jan A, et al. Quantitative study on the trade and ecological aspect of spice plants in the markets of district bannu, khyber pakhtunkhwa, Pakistan. Ethnobotany Research and Applications. 2021;22. | Non-human study |
|  | Shahbaz A, Parizad N. “It's like the snakes and ladders game.”; lived experience of patients with multiple sclerosis regarding their return to work: A qualitative study. Current Psychology. 2023;42(21):18255-67. | Non-human study |
|  | Shahrajabian MH, Sun WL. Iranian Traditional Medicine (ITM) and Natural Remedies for Treatment of the Common Cold and Flu. Reviews on Recent Clinical Trials. 2024;19(2):91-100. | Non-human study |
|  | Sivan N, Werner YL. Revision of the middle-eastern dwarf-snakes commonly assigned to eirenis coronella (colubridae). Zoology in the Middle East. 2003;28(1):39-59. | Non-human study |
|  | Soleimani G, Shahri ES, Shahraki N, Godarzi F, Mousavi SHS, Tavakolikia Z. Clinical and Laboratory Findings and Prognosis of Snake and Scorpion Bites in Children under 18 Years of Age in Southern Iran in 2018-19. International Journal of Pediatrics-Mashhad. 2021;9(1):12795-804. | Non-human study |
|  | Taghavizad R. The Emergence of a New Record of Scincella lateralis Under the Canopy Layer of Ligustrum vulgare in Iran and the Similarity of the Ecological Niche with America. Ecopersia. 2024;12(3):233-45. | Non-human study |
|  | Valdiani A, Kadir MA, Tan SG, Talei D, Abdullah MP, Nikzad S. Nain-e Havandi Andrographis paniculata present yesterday, absent today: a plenary review on underutilized herb of Iran's pharmaceutical plants. Molecular Biology Reports. 2012;39(5):5409-24. | Non-human study |
|  | Kassiri H, Dehghani R, Khodkar I, Ahmad FA, Kassiri A. Epidemiological profile of venomous animal bites and stings (A neglected problem) in the north of Sistan - Baluchistan province, South-Eastern Iran. Journal of Entomological Research. 2022;46(4):917-21. | - |
|  | Dehghani R, Varzaneh AA, Varzandeh M, Akbari M, Jahani A. Venomous animal bites and stings in Lordegan city, Chaharmahal Bakhtiari province of Iran in 2019-2020. Journal of Entomological Research. 2022;46(3):694-8. | - |
|  | Nejadrahim R, Sahranavard M, Aminizadeh A, Delirrad M. Research paper: Snake envenomation in north-west Iran: A three-year clinical study. International Journal of Medical Toxicology and Forensic Medicine. 2019;9(1):31-8. | - |
|  | Kassiri H, Khodkar I, Kazemi S, Kasiri N, Lotfi M. Epidemiological analysis of snakebite victims in southwestern Iran. Journal of Acute Disease. 2019;8(6):260-4. | - |
|  | Kassiri H, Tandis F, Lotfi M. Incidence and epidemiological profile of snakebites and scorpion stings in northern Khuzestan province, southwestern Iran: A descriptive, analytical study. Asian Journal of Pharmaceutics. 2018;12(3):S888-S95. | - |
|  | Ebrahimi V, Hamdami E, Khademian MH, Moemenbellah-Fard MD, Vazirianzadeh B. Epidemiologic prediction of snake bites in tropical south Iran: Using seasonal time series methods. Clinical Epidemiology and Global Health. 2018;6(4):208-15. | - |
|  | Dehghani R, Fathi B, Shahi MP, Jazayeri M. Ten years of snakebites in Iran. Toxicon : official journal of the International Society on Toxinology. 2014;90:291-8. | - |
|  | Dehghani R, Dadpour B, Mehrpour O. Epidemiological profile of snakebite in Iran, 2009-2010 based on information of ministry of health and medical education. International Journal of Medical Toxicology and Forensic Medicine. 2014;4(2):33-41. | - |

After removing duplicate articles, 195 articles remained, of which 166 were excluded due to reasons such as non-human studies (n=156), unavailability of full text (n=2), review articles (n=5), and case reports (n=3). The remaining 29 articles were selected for full text review, of which 21 studies were excluded due to review articles, unclear sample size, lack of precise population definition, and failure to report incidence. Finally, 8 articles were selected for data extraction and statistical analysis.

The blue row contains 21 articles that were reviewed for full text and then removed.

The green rows contain 8 articles that were selected for data extraction.
